# Why do pressure injuries still occur? A multicenter qualitative study of nurses and caregivers

**DOI:** 10.1016/j.jdin.2024.05.006

**Published:** 2024-07-23

**Authors:** Wilson Sim, Tan Hwei Sian Clara Michelle, Nur Qasrina Binte Iskandar Lim, Veronica Loh, Cheryl Wan Xuan Chua, Jason Er, Joyce Er, Phillip Phan, Ellie C.E. Choi

**Affiliations:** a Yong Loo Lin School of Medicine, National University of Singapore, Singapore, Singapore; b Department of Medicine, National University Hospital, Singapore, Singapore; c Alexandra Hospital, National University Health System, Singapore, Singapore; d Carey Business School, Johns Hopkins University, Baltimore, Maryland; e Medicine, Johns Hopkins Medicine, Baltimore, Maryland; f Division of Dermatology, Department of Medicine, National University Hospital, Singapore, Singapore; g Centre for research in Health System Performance, Yong Loo Lin School of Medicine, National University of Singapore, Singapore, Singapore

**Keywords:** barriers, bedsore, facilitators, health services research, patient-centered care, pressure injury, pressure ulcer, preventive health

## Abstract

**Background:**

The frequent occurrence of pressure injuries despite their preventability raises important questions about our understanding of the barriers to care. This study explores the lived experience of nurses and caregivers in Singapore to establish a conceptual framework for better understanding of pressure injuries arising in the community.

**Methods:**

A multicenter qualitative study was conducted utilizing semistructured interviews and focused group discussions of nurses and caregivers of patients with pressure injuries. Data were collected with a narrative inquiry approach and analyzed with grounded theory. An iterative cycle of interviewing, coding, discussion, and reflection was conducted until theoretical saturation.

**Results:**

Ten inpatient nurses and 10 caregivers from the community were recruited. Analysis identified cognitive (awareness and caregiver training), emotional (motivation and empowerment), resource (human and infrastructural), and biomedical factors which collectively impacted the effectiveness of prevention. Additionally, constructs of learning and sustainability of caregiving played a crucial role in long term prevention.

**Limitation:**

The derived framework requires further validation with quantitative data and may not be generalizable to other cultural and economic populations.

**Conclusion:**

Multiple constructs have been identified to have a synergistic effect in prevention. Targeted measures should be implemented by healthcare institutions to better equip caregivers in prevention.

**Capsule Summary**

• The study pinpoints cognitive, emotional, resource, and biomedical factors as reasons for the widespread prevalence of preventable pressure injuries.
• It suggests a synergistic approach involving salient education about complications, enhancement of the learning feedback loop, and measures to enhance sustainability.

## Introduction

Pressure injuries, also known as pressure ulcers or bed sores, arise from continuous pressure causing localized skin and soft tissue damage. Pressure injuries are common, with a prevalence between 5% and 30% in hospitalized patients,^1^, ^2^, ^3^, ^4^ and accounts for up to 60,000 fatalities yearly, with mortality rates 2 to 6 times greater than other illnesses.^3^ Additionally, the demanding wound healing process creates associated caregiver depression and stress.^5^ They also contribute to a financial burden, where for example in 2016, the expenditures associated with hospital-acquired pressure injuries in the United States alone exceeded $26.8 billion.^6^

Formulated guidelines by the National Pressure Injury Advisory Panel and the European Pressure Ulcer Advisory Panel^7^ are centered upon key areas including nutrition, frequent repositioning, skin hygiene and hydration, managing incontinence and utilizing pressure-relief resources.^8^ Pressure injury prevention guidelines in Singapore are additionally augmented with caregiver training to equip caregivers with the knowledge and skills to better manage the care of their loved ones at home.^9^^,^^10^ Government subsidies further support this initiative by facilitating the procurement of essential resources.^11^

Despite these efforts, a significant proportion of pressure injuries develop in the community with a reported prevalence of 3% to 11%.^12^, ^13^, ^14^, ^15^, ^16^ A study in New England revealed that 70.6% of hospitalized patients with pressure injuries had developed them at home.^17^

These highlight a significant gap between optimal and actual practices.

In households, caregivers of patients at risk of pressure ulcers include family members and domestic workers. Domestic workers are migrant non-healthcare professionals who live with employers and aid with household chores and provide care.^18^ They thus assume a critical role in pressure injury prevention and management in the community.

This paper investigates the lived experience of caregivers and nurses in Singapore to construct a conceptual framework. This framework aims to support tailored interventions, bridging the gap between clinical guidelines and practical, in-home care for pressure injury prevention and management.

## Methods

This is a multicenter qualitative study of caregivers and nurses across 3 tertiary hospitals in Singapore (National University Hospital, Alexandra Hospital, and Ng Teng Fong General Hospital). Caregivers of a patient at risk of pressure injury (eg chair-bound or bedridden patients for at least 6 months) or nurses with more than a year of clinical experience and having cared for at least 1 patient with pressure injury were recruited. Nurses work closely with hospitalized patients and their caregivers. Thus, their insights and perspectives into the barriers and facilitators of care were sought. Nurses and caregivers were recruited through word-of-mouth. Semistructured (in 1:1 interviewer/interviewee ratio) or focused group interviews (in 2:5 ratio) were conducted in English by W.S, C.M.T, and E.C with a narrative inquiry approach.

Interviews were audio-recorded and transcribed verbatim. Data analysis was performed using grounded theory.^19^ Descriptive open coding was first performed using line-by-line and incident coding, followed by analytic focused coding wherein codes were compared to each other. Similar codes were then categorized into higher ordered themes through axial coding and organized into a framework. Analysis was performed by W.S., C.M.T, and E.C. using ATLAS.ti 8.0.9.^20^ The final framework was unanimously approved by all authors. Modifications to the interview guide were made to reflect initial learnings and to probe findings of interest (Supplementary Materials 1 and 2, available via Mendeley at https://data.mendeley.com/datasets/zfcsvvwghm/1). The cycle of interviewing, coding, discussion, and reflection was continued iteratively until theoretical saturation, which previous qualitative dermatological studies suggest this to occur with 10-30 participants.

All interviewers were trained in gathering qualitative information and maintained a high level of sensitivity to their roles as coconstructors of meaning. The diligent writing of memos and reflexive documentation were emphasized to guard against preconceived notions and biases. The study was designed and reported following the Consolidated Criteria for Reporting Qualitative Research guidelines^21^ (Supplementary Material 4, available via Mendeley at https://data.mendeley.com/datasets/zfcsvvwghm/1) and in accordance with Morse qualitative research principles.^22^ The study was approved by the National Healthcare Group’s Domain Specific Review Board (reference 2022/00470).

## Results

Two focused group discussions and 8 interviews were conducted with 10 nurses and 10 caregivers between February and July 2023. All interviews were conducted in person with an average interview time of 48 minutes. Demographics of the caregivers and nurses are presented in Tables I and II respectively.

**Table I:** Demographics of caregivers and patients (*n* = 10)

Age (years)	
Mean (SD)	56 (13)
Sex	
Male	1 (10%)
Female	9 (90%)
Relationship to caregiver	
Daughter	6 (60%)
Spouse	3 (30%)
Domestic worker	1 (10%)
Number of families with domestic worker	6 (75%)
Age of patient (years)	
Mean (SD)	76 (11)
Mobility of patient	
Bedridden	7 (87.5%)
Chair-bound	1 (12.5%)
Continence status of patient	
Continent	2 (25%)
Single incontinence on diapers	4 (50%)
Dual incontinence on diapers	2 (25%)
Weight of patient (kg)	
Mean (SD)	58 (13)
History of pressure injury	
None	1 (12.5%)
One	5 (62.5%)
Two	2 (25%)

*SD*, Standard deviation.

Analysis via grounded theory^19^ first identified 6 broad themes. These include the cognitive, emotional, resource, and biomedical factors impact the effectiveness of prevention strategies, and sustainability of caregiving and learning which determine the long-term equilibrium (Table III and Supplementary Material 3, available via Mendeley at https://data.mendeley.com/datasets/zfcsvvwghm/1). These themes were then organized into an overarching framework wherein the relationship between various contributory factors including a reinforcing feedback loop is highlighted.

**Table II:** Demographics of nurses (*n* = 10)

Age (years)	
Mean (SD)	35 (10)
Sex	
Male	1 (10%)
Female	9 (90%)
Years of experience	
Mean (SD)	10 (7)
Appointment	
Senior staff nurse	8 (80%)
Assistant nurse clinician	2 (20%)

*SD*, Standard deviation.

**Table III:** Summary of codes, primary, secondary themes, and proposed solutions in pressure injury prevention

Theme	Description	Proposed solutions
Cognitive		
Awareness	Awareness of prevention, susceptibility, early signs, complications and wound care	1. Extend education strategies beyond the institution to the community and population level 2. Improve effectiveness of education - Start education early - Susceptibility and potential extent of complications may be emphasized through sharing of experiences from other patients of a similar profile - Preparation not only for current care needs, but potential changes or complications that may occur in the future - Hands on training i.e. relevant to the home environment - Harnessing digital tools that are more effective than traditional educational material
Caregiver training	Focus on pressure injury prevention, timing, involvement of all stakeholders, building self-efficacy	
Emotional		
Motivation	Intrinsic motivation, prioritization of prevention	1. Motivate caregivers with negative consequences of pressure injuries and benefits of being pressure-injury free - Some nurses reported efficacy of “scare tactics” 2. Ongoing encouragement for the caregiver, whether by the healthcare team or family members 3. Facilitating and encouraging collaboration and self-efficacy 4. Setting realistic goals for wound recovery
Empowerment	Empowerment from within, empowerment by others	
Resource		
Human resource	Sufficiency of manpower, quality of relationship, involvement of family members	1. Improve both availability and awareness of resources 2. Improve training of domestic workers to reduce changeover, promotes trust and better care 3. Restructuring the physical environment and setting up routines 4. Ongoing development of pressure injury prevention strategies that overcome barriers such as the need to physical turn the patient
Infrastructural resource	Accessibility of preventive resources and wound dressing materials, home environment	
Biomedical state of patient		
Biomedical state of patient	Direct risk factors (pre-existing skin condition, incontinence, degree of immobility) and indirect factors (nutrition, psychological state and cooperativeness, pain)	1. Optimize risk factors such as incontinence, contractures, poor nutrition 2. Effectively manage behavioral issues whether through psycho-behavioral and pharmacological strategies.
Sustainability		
Sustainability	Physical (ease of medical access, physical respite) Emotional (caregiver stress and burnout,expectations regarding chronicity) Financial (ability to finance required care)	1. Offering adaptive remote and digital tele-support solutions, tailored to the specific challenges caregivers face 2. Improve availability of home and community medical and nursing services
Learning		
Learning	From one’s own experience From others e.g. nurses, physicians, educational programmes	1. Continual reinforcement of prevention by healthcare team at multiple touchpoints 2. Enhance collective knowledge and practical skills through peer-learning and self-reflection

## Cognitive

### Awareness

*“Because I did not know it will become such a big wound with infection, we would take action earlier if we had more knowledge of this thing (pressure injury)”*

—Mdm AHP, caregiver

Caregivers often lack sufficient awareness on the true susceptibility and potential complications of pressure injuries until an advanced pressure injury had already developed. The implications of pressure injuries such as the cost of caring for pressure injuries being “*more expensive”* and *“more difficult”* than preventing them should be emphasized to caregivers. Second, hospitals may underestimate the risk of pressure injuries (patients *“deemed as low risk”*) leading to the inadequate preparation of caregivers. Finally, acute changes in risk factors led to caregivers feeling unprepared to deal with the risk.

### Caregiver training

*“I think the hospital should have actually done more… we did not realize that (pressure injury) would come so fast.”*

*—*Ms L, caregiver

Problems in this domain include pressure injury training being quickly conducted “*one or 2 days before discharge”* without knowing if the learning objectives were achieved. One caregiver mentioned that she “didn't really learn very much about the wound caring until (she became) hands on”. In Singapore where the main caregiver is often a foreign domestic worker, it was crucial to *“always best to educate both the main spokesperson (family member) and the (domestic worker) at the same time”* to ensure consistent and concordant care. Finally, nurses identified a discrepancy between what was taught in the hospital and what could be implemented at home. For example, the availability of instrument tables, sterile sets, and additional assistance to turn the patient adequately were challenging to replicate at home.

## Emotional

### Motivation

Motivation amongst caregivers were intrinsic and *“boils down to the love of the family members”*. For nonfamily caregivers, motivational levels were varied resulting in some care being excellent and others being unsatisfactory.

*“He's the most important because he has a problem with his body, the household chores can be done later when everything is ok for him”*

—Mr TGS’s domestic worker and caregiver, referring to her job responsibilities.

The patient’s motivation was also important. While some were “*very motivated”,* others may be uncooperative and refuse to eat or be turned. One caregiver responded to her father’s tantrum by saying *“since you don’t want to eat, I also won't eat”*, making him *“reluctantly eat”*.

### Empowerment

*“I've experienced people telling me, it's your job (to look after my family). And of course, there are also people telling me you have done a good job - the feeling is different.”*

—Ms D, caregiver

Addressing the *"knowledge deficit”* empowers caregivers to act by increasing their confidence and resilience to the ongoing challenges of identifying and responding to pressure injuries. Additionally, witnessing improvements in their patients' conditions and receiving the encouragement from the medical team with words like, *"gradually it will be better"* were other sources of empowerment.

## Resources

### Human resource

Caregivers and nurses cite labor shortages as a major challenge in patient care, particularly for older caregivers. For instance, 1 caregiver had to *“wait for (his) domestic (worker) to come back at 6 o'clock to change (his wife’s diapers)”*.

Balancing trust with and oversight of domestic workers is crucial. *“I don't treat her as a* (domestic worker)*, we treat her as one of my family, she eats what we eat… so I think she is also very happy working with us.” Conversely,* when the family is not cohesive, they may *“throw the patient to the helper and the helper doesn't do much”* leading to the suboptimal care of the patient.

### Infrastructure

*“A family that is well-off can buy the best equipment and they are more available* [have more capacity] *to take care of the patient. They can even hire a helper for this patient.”*

—Ms L, caregiver

While the procurement of preventive and wound care resources was greatly aided by government subsidies, the financial strain was still present for many, such as those in the "sandwich income" bracket who do not qualify for government subsidies because they earn just above the threshold.

## Biomedical state of the patient

*“Yeah, that's why he was admitted and being treated for multiple things. So one thing is his urinary tract infection and also his bed sore. So he got more complex reasons as to why he was being hospitalized”*

—Ms J, caregiver

The presence of multiple medical issues represented a distraction from pressure injury prevention. Comorbidities such as “*Parkinson’s disease, dementia”* and pain also limited the ability of patients to express themselves and to cooperate with prevention efforts.

## Sustainability of caregiving

*“It's like I don't have a life outside work, my own housework, and to take care of my mother. So it's just these three things in my life. Other than that, I don't really have a social life.”*

—Ms CY, caregiver

Burnout may occur from a life consumed by caring for the patient. Some caregivers who felt “*lost”* appreciated ongoing medical support through the “*ever-changing nature”* of pressure injuries. Another crucial aspect of coping involved accepting that “*even with all the effort put into pressure injury prevention, it may not always go in the way you expect it to*.”

## Learning

Caregiving is a dynamic process marked by the evolution of attitudes and practices. Pressure injury management involves acquiring factual knowledge *“I've got more knowledge, so I was able to know when it is being infected…”* and figuring out creative and adaptive strategies for individual patients’ needs. For example, 1 of the caregivers made modifications to their own shower chair by *“putting a soft cushion over the shower chair so it is not too painful for him”*.

Learning also occurred through external influences, “*We also see how the nurses at XX Hospital do it as he was hospitalized numerous times. When we visit him every day, we actually requested them to teach us.” –* Ms M, caregiver.

## Framework

**Fig 1:**
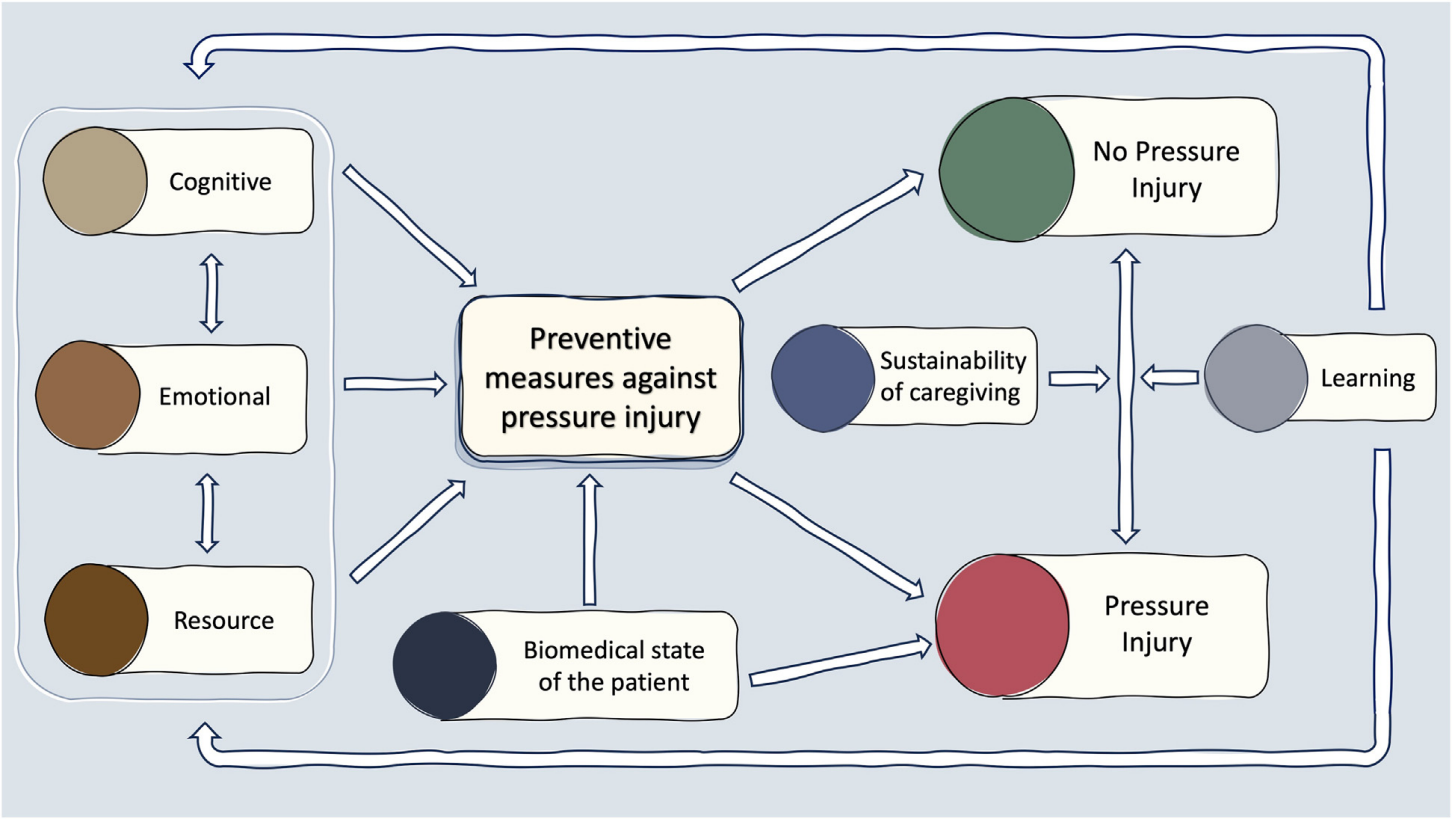
Framework of constructs impacting pressure injury prevention.

From the themes, we derived a framework to elucidate the nuanced process of preventing pressure injuries (Fig 1).

Illustrative case: Mdm AHP became caregiver to her newly bedridden father. She had insufficient cognitive awareness and resources dedicated to pressure ulcer prevention which led to her father readmission for an infected stage 4 pressure injury months after discharge. In an institutional care facility, his pressure ulcer healed, but this did not translate to sufficient improvement in preventive measures at home which resulted in a recurrence of the pressure injury. In contrast to the first episode, this second episode carried a sense of shock and guilt for Mdm AHP, fueling a stronger commitment to effective learning. She began using resources like pressure-relief mattresses and adopted greater responsibility rather than relegating care to health care workers or the domestic worker. Positive reinforcement from the health care team and observational learning from home care nurses enhanced the learning loop. Despite the observed success, the recovery process remained fraught with challenges, underscoring the need for ongoing support.

## Proposed solutions

Proposed solutions were formulated from nurses, patients, team discussion, and integrated with insights from a literature review (Table III). The health belief^23^ and COM-B model^24^ were used to guide these suggestions. Notable suggestions include instituting pressure injury prevention education at a population level (similar to education for breast cancer screening, dengue, and vein thrombosis prevention). This could be achieved through various channels such as mass media and community outreach talks, which have proven effective in raising awareness for conditions like cancer screening and dengue.^25^ The training and availability of home care nurses and teleconsultations can be enhanced to improve care and promote sustainability.^26^^,^^27^ Individually, the health care team of doctors and nurses play a pivotal role in caregiver education (including the judicious use of scare tactics) to inspire commitment to prevention.

## Discussion

This study explored the lived experiences of caregivers and nurses to comprehensively identify the factors influencing pressure injury prevention at home.

The complexity of inter-related factors shows why addressing factors in isolation will be unsuccessful. For example, a caregiver who may be financially endowed still needs to be involved in active prevention to avoid over-reliance on resources such as pressure-relief mattresses or domestic workers. Efforts to improve pressure injury prevention can also be synergistic. Empowerment by health care teams can lead caregivers to proactively seek knowledge and improve their skills. Supporting caregivers at various touch points with information relevant to the patient’s current stage would enhance the learning process and ensure sustainability.

The context of Singapore’s health care system should be considered to gauge the relevance and generalizability of findings. Singapore has a mixed system comprising heavily subsidized public care which is utilized by most individuals, alongside private health care. In our study, most patients benefited from government subsidies, mitigating financial constraints. Studies in other context may elicit a financial constraint as a greater barrier.

A limitation of this study as with qualitative studies in general is the assumption that participants have good insight into their own thoughts and behaviors, which may not necessarily be true. However, an iterative process of interviewing, coding, discussion, and reflection was used to triangulate perspectives from different informants to mitigate this limitation.

A notable gap in current literature is the scarcity of implementation studies, especially in the context of pressure injury prevention in home and community settings. The few implementation studies have examined practices within health care institutions.^28^, ^29^, ^30^ These studies identified more commonly adopted (eg daily skin assessment, pressure redistribution) and less frequently implemented measures (nutrition support, moisturizer management).^29^ Our study has 2 implications with regard to implementation. First, implementation requires an understanding of the barriers to success. An intervention (such as turning a patient frequently) may work in hospital setting but will have to be modified to account for the lack of manpower or capability at home. Second, education and clinician support are needed to scaffold any interventions in the home. The form these take will depend on the available technology, ability of caregivers, and their willingness to use them. More generally, future research should focus on implementational strategies and barriers of pressure injury prevention in a home setting.

Our study highlighted the need for the early identification of susceptible patients in the hospital, which can potentially be achieved through the combination of artificial intelligence-based image recognition technologies and predictive risk stratification models.^31^ This step is crucial for directing resources, such as clinician support, more effectively toward those with greater susceptibility. Additionally, having a scoring system to identify susceptible patients can also motivate the healthcare team to provide rigorous training.

Finally, we call for further quantitative studies to objectively quantify the relative impact of the different constructs we identified in this study on the success of prevention efforts to guide the development of interventions.

## Conclusion

While caregivers are the primary and key players in the management of pressure injuries, their ability to successfully prevent pressure injuries are reliant on various external factors including health care professionals, community and government support. In this study, we have identified multiple constructs and highlighted the importance of these constructs working synergistically together to prevent pressure injuries at home.

## Patient and public involvement

Our patient and public involvement representatives (Mdm AHP, Ms J Ang, and Ms L Ang) were caregivers. They participated in qualitative interviews and contributed to theme development, reviewed the framework and provided suggestions for improving prevention against pressure injuries. Additionally, they reviewed and approved the final manuscript.

Access to data: The principal investigator E.C. had full access to all the data in the study and takes responsibility for the integrity of the data and the accuracy of the data analysis.

Data sharing: Deidentified data supporting the findings of this study are available on reasonable request from the corresponding authors W.S. and E.C.

## Conflicts of interest

None disclosed.

## References

[1] Graves N., Maiti R., Aloweni F.A.B., Yuh A.S., Lo Z.J., Harding K. (2020). Pressure injuries among admissions to a hospital in the tropics. Int Wound J.

[2] Li Z., Lin F., Thalib L., Chaboyer W. (2020). Global prevalence and incidence of pressure injuries in hospitalised adult patients: a systematic review and meta-analysis. Int J Nurs Stud.

[3] Afzali Borojeny L., Albatineh A.N., Hasanpour Dehkordi A., Ghanei Gheshlagh R. (2020). The incidence of pressure ulcers and its associations in different wards of the hospital: a systematic review and meta-analysis. Int J Prev Med.

[4] Sardo P.M.G., Teixeira J.P.F., Machado A.M.S.F., Oliveira B.F., Alves I.M. (2023). A systematic review of prevalence and incidence of pressure ulcers/injuries in hospital emergency services. J Tissue Viability.

[5] Roussou E., Fasoi G., Stavropoulou A. (2023). Quality of life of patients with pressure ulcers: a systematic review. Med Pharm Rep.

[6] Padula W.V., Delarmente B.A. (2019). The national cost of hospital-acquired pressure injuries in the United States. Int Wound J.

[7] Kottner J., Cuddigan J., Carville K. (2019). Prevention and treatment of pressure ulcers/injuries: the protocol for the second update of the international Clinical Practice Guideline 2019. J Tissue Viability.

[8] Pressure injury prevention measures: overview of systematic reviews - PMC. https://www.ncbi.nlm.nih.gov/pmc/articles/PMC10742601/.

[9] MOH | guidelines. https://www.moh.gov.sg/hpp/nurses/guidelines/GuidelineDetails/cpgnursing_prediction_prevention_pressure_ulcers_adults.

[10] Chan E.Y., Wu L.T., Ng E.J.Y., Glass G.F., Tan R.H.T. (2022). Applying the RE-AIM framework to evaluate a holistic caregiver-centric hospital-to-home programme: a feasibility study on Carer Matters. BMC Health Serv Res.

[11] ACE technology guidances. https://www.ace-hta.gov.sg/healthcare-professionals/ace-technology-guidances.

[12] Rodrigues A.M., Ferreira P.L., Ferré-Grau C. (2016). Providing informal home care for pressure ulcer patients: how it affects carers' quality of life and burden. J Clin Nurs.

[13] ALFadhalah T., Lari M., Al Salem G., Ali S., Al Kharji H., Elamir H. (2024). Prevalence of pressure injury on the medical wards of public general hospitals in Kuwait: a national cross-sectional study. BMC Health Serv Res.

[14] Sari S.P., Everink I.H., Sari E.A. (2019). The prevalence of pressure ulcers in community-dwelling older adults: a study in an Indonesian city. Int Wound J.

[15] Gunningberg L., Stotts N.A., Idvall E. (2011). Hospital-acquired pressure ulcers in two Swedish County Councils: cross-sectional data as the foundation for future quality improvement. Int Wound J.

[16] Kirkland-Khyn H., Teleten O., Joseph R., Maguina P. (2019). A descriptive study of hospital- and community-acquired pressure ulcers/injuries. Wound Manag Prev.

[17] Corbett L.Q., Funk M., Fortunato G., OʼSullivan D.M. (2017). Pressure injury in a community population: a descriptive study. J Wound Ostomy Continence Nurs.

[18] Rozario P.A., Hong S.I. (2019). Foreign domestic workers and eldercare in Singapore: who hires them?. J Aging Soc Policy.

[19] Charmaz K. (2015).

[20] ATLAS.ti Scientific Software Development GmbH (2023). https://atlasti.com.

[21] Tong A., Sainsbury P., Craig J. (2007). Consolidated criteria for reporting qualitative research (COREQ): a 32-item checklist for interviews and focus groups. Int J Qual Health Care.

[22] Morse J.M. (2015). Critical analysis of strategies for determining rigor in qualitative inquiry. Qual Health Res.

[23] Rosenstock I.M. (1990). Health behavior and health education: theory, research, and practice.

[24] Michie S., van Stralen M.M., West R. (2011). The behaviour change wheel: a new method for characterising and designing behaviour change interventions. Implement Sci.

[25] Grilli R., Ramsay C., Minozzi S. (2002). Mass media interventions: effects on health services utilisation. Cochrane Database Syst Rev.

[26] Sim W., Choi E., Chandran N.S. (2023). Where are we with teledermatology? Two years in the wake of COVID-19. JMIR Dermatol.

[27] Choi E.C.E., Heng L.W., Tan S.Y., Phan P., Chandran N.S. (2022). Factors influencing use and perceptions of teledermatology: a mixed-methods study of 942 participants. JAAD Int.

[28] Wan C.S., Cheng H., Musgrave-Takeda M. (2023). Barriers and facilitators to implementing pressure injury prevention and management guidelines in acute care: a mixed-methods systematic review. Int J Nurs Stud.

[29] Edsberg L.E., Cox J., Koloms K., VanGilder-Freese C.A. (2022). Implementation of pressure injury prevention strategies in acute care: results from the 2018-2019 international pressure injury prevalence survey. J Wound Ostomy Continence Nurs.

[30] Scovil C.Y., Flett H.M., McMillan L.T. (2014). The application of implementation science for pressure ulcer prevention best practices in an inpatient spinal cord injury rehabilitation program. J Spinal Cord Med.

[31] Lau C.H., Yu K.H.O., Yip T.F. (2022). An artificial intelligence-enabled smartphone app for real-time pressure injury assessment. Front Med Technol.

